# Bis[2-(3-chloro­benzyl­idene)propanoato-κ^2^ *O*,*O*′]diethyl­tin(IV)

**DOI:** 10.1107/S1600536808018321

**Published:** 2008-06-21

**Authors:** Niaz Muhammad, M. Nawaz Tahir, Saqib Ali, Zia-ur-Rehman

**Affiliations:** a Department of Chemistry, Quaid-i-Azam University, Islamabad 45320, Pakistan; b University of Sargodha, Department of Physics, Sagrodha, Pakistan

## Abstract

In the mol­ecule of the title compound, [Sn(C_2_H_5_)_2_(C_10_H_8_ClO_2_)_2_], the Sn atom is six-coordinated in a distorted tetra­gonal–bipyramidal configuration by four O atoms in the equatorial plane and two C atoms in the axial positions. Intra­molecular C—H⋯O hydrogen bonds result in the formation of two planar and two non-planar five-membered rings; the latter adopt envelope conformations. There are weak π–π inter­actions between aromatic rings, with centroid-to-centroid distances of 3.796 (2) and 4.171 (2) Å. There is also a single C—Cl⋯π inter­action [C—Cl = 1.740 (4), Cl⋯π = 3.795 (2) C⋯π = 3.697 (4) Åand C—Cl⋯\p =73.45 (11)°].

## Related literature

For general background, see: Xie *et al.* (1996 ▶); Nath *et al.* (2001 ▶); Crowe (1989 ▶); Gielen *et al.* (2000 ▶). For related literature, see: Hanif *et al.* (2007 ▶); Parvez *et al.* (1997 ▶).
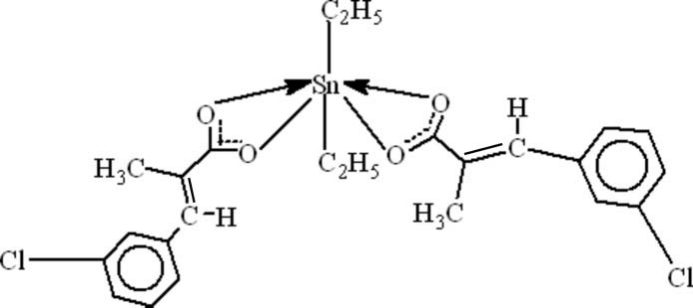


## Experimental

### 

#### Crystal data

[Sn(C_2_H_5_)_2_(C_10_H_8_ClO_2_)_2_]
*M* *_r_* = 568.04
Triclinic, 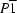

*a* = 7.5171 (3) Å
*b* = 12.8388 (5) Å
*c* = 12.8712 (5) Å
α = 98.724 (2)°
β = 92.250 (2)°
γ = 100.148 (2)°
*V* = 1205.84 (8) Å^3^
*Z* = 2
Mo *K*α radiation
μ = 1.31 mm^−1^
*T* = 296 (2) K
0.25 × 0.18 × 0.15 mm

#### Data collection

Bruker Kappa APEXII CCD diffractometer
Absorption correction: multi-scan (*SADABS*; Bruker, 2005 ▶) *T*_min_ = 0.756, *T*_max_ = 0.819
20475 measured reflections
4718 independent reflections
4364 reflections with *I* > 2σ(*I*)
*R*_int_ = 0.030

#### Refinement

*R*[*F* ^2^ > 2σ(*F* ^2^)] = 0.027
*wR*(*F* ^2^) = 0.090
*S* = 1.23
4718 reflections
281 parameters
H-atom parameters constrained
Δρ_max_ = 1.19 e Å^−3^
Δρ_min_ = −0.70 e Å^−3^

### 

Data collection: *APEX2* (Bruker, 2007 ▶); cell refinement: *APEX2*; data reduction: *SAINT* (Bruker, 2007 ▶); program(s) used to solve structure: *SHELXS97* (Sheldrick, 2008 ▶); program(s) used to refine structure: *SHELXL97* (Sheldrick, 2008 ▶); molecular graphics: *ORTEP-3 for Windows* (Farrugia, 1997 ▶); software used to prepare material for publication: *WinGX* (Farrugia, 1999 ▶).

## Supplementary Material

Crystal structure: contains datablocks global, I. DOI: 10.1107/S1600536808018321/hk2473sup1.cif

Structure factors: contains datablocks I. DOI: 10.1107/S1600536808018321/hk2473Isup2.hkl

Additional supplementary materials:  crystallographic information; 3D view; checkCIF report

## Figures and Tables

**Table:** 

Sn—C1	2.110 (3)
Sn—C3	2.113 (4)
Sn—O3	2.1342 (19)
Sn—O1	2.137 (2)
Sn—O2	2.477 (2)
Sn—O4	2.556 (2)

**Table:** 

C1—Sn—C3	154.28 (15)
C1—Sn—O3	98.88 (12)
C3—Sn—O3	101.22 (13)
C1—Sn—O1	98.94 (11)
C3—Sn—O1	99.02 (12)
O3—Sn—O1	83.85 (8)
C1—Sn—O2	86.19 (12)
C3—Sn—O2	88.69 (13)
O3—Sn—O2	139.87 (8)
O1—Sn—O2	56.10 (7)
C1—Sn—O4	89.58 (11)
C3—Sn—O4	89.11 (12)
O3—Sn—O4	54.58 (7)
O1—Sn—O4	138.42 (7)
O2—Sn—O4	165.46 (7)

**Table 2:** Hydrogen-bond geometry (Å, °)

*D*—H⋯*A*	*D*—H	H⋯*A*	*D*⋯*A*	*D*—H⋯*A*
C7—H7*A*⋯O2	0.96	2.31	2.780 (5)	109
C8—H8⋯O1	0.93	2.30	2.736 (3)	108
C17—H17*A*⋯O3	0.96	2.31	2.749 (4)	107
C18—H18⋯O4	0.93	2.37	2.785 (3)	107

**Fig. 1.**
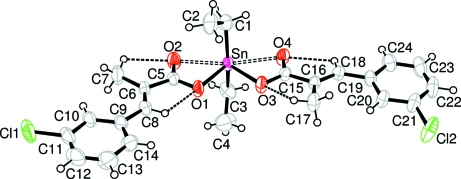
The molecular structure of the title molecule, with the atom-numbering scheme. Displacement ellipsoids are drawn at the 50% probability level. Hydrogen bond is shown as dashed line.

**Fig. 2.**
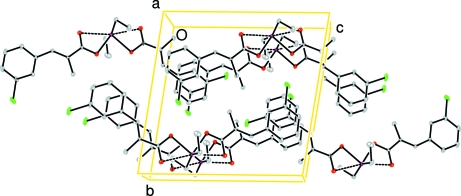
A packing diagram of (I).

## supplementary crystallographic information

### Comment

Organotin compounds have attracted much interest owing to their potential use in industry and agriculture (Xie *et al.*, 1996; Nath *et al.*, 2001). In the pharmaceutical industry, a number of dialkyltin carboxylate derivatives are being used as efficient antitumor and anticancer agents (Crowe, 1989; Gielen *et al.*, 2000). In continuation of our studies on the structural aspects of organotin(IV) carboxylates, we report herein the crystal structure of the title compound, (I).

In the molecule of (I) (Fig. 1), the Sn atom is six-coordinated in distorted tetragonal bipyramidal configuration (Table 1) by four O atoms in the equatorial plane and two C atoms in the apical positions. The bond lengths and angles are within normal ranges, which are comparable with the corresponding values in bis(3,4-methylenedioxybenzoyl)diethyltin(IV), (II) (Hanif *et al.*, 2007) and diethylbis[3-(2-thienyl)-2-propenoato-*O*,*O*']tin(IV), (III) (Parvez *et al.*, 1997). The Sn—C1 [2.110 (3) Å] and Sn—C3 [2.113 (4) Å] bonds in (I) are reported as 2.137 (6) and 2.138 (7) Å in (II) and 2.155 (2) Å in (III). On the other hand, the Sn—O bonds are in the range of [2.1342 (19)–2.556 (2) Å] in (I). They are reported as in the ranges of [2.142 (4)–2.544 (4) Å] in (II) and [2.105 (5) and 2.538 (6) Å] in (III).

Rings *A* (Sn/O1/O2/C5), *B* (Sn/O3/O4/C15), *C* (C9–C14) and *D* (C19–C24) are, of course, planar, and the dihedral angles between them are *A*/*B* = 3.05 (11)°, *A*/*C* = 2.10 (12)°, *A*/*D* = 1.58 (10)°, *B*/*C* = 1.73 (12)°, *B*/*D* = 4.41 (11)° and *C*/*D* = 3.68 (13)°. So, they are nearly coplanar. The intramolecular C—H···O hydrogen bonds (Table 2) result in the formation of two planar and two non-planar five-membered rings: *E* (O1/C5/C6/C8/H8), *F* (O4/C15/C16/C18/H18), *G* (O2/C5–C7/H7*A*) and *H* (O3/C15–C17/H17*A*), respectively. Rings *G* and *H* adopt envelope conformations, with H7*A* and H17*A* atoms displaced by 0.184 and 0.356 Å from the planes of the other ring atoms, respectively.

In the crystal structure, the molecules are elongated along the *c* axis and stacked along the *a* axis (Fig. 2). The weak π–π interactions between aromatic rings *CgC*···*CgD*^i^ and *CgC*···*CgD*^ii^ [symmetry codes: (i) *x*, *y*, *z* - 1 and (ii) *x* + 1, *y*, *z* - 1] may be effective in the stabilization of the structure, with centroid–centroid distances of 3.796 (2) and 4.171 (2) Å, respectively. There is also a single C—Cl···π interaction, C21—Cl2···*CgC*^iii^ [symmetry code: (iii) *x* - 1, *y*, *z* + 1], at a distance of 3.797 (2) Å.

### Experimental

The title compound (I), was prepared by the reaction of stoichiometric amounts of the sodium 3-(3-chlorophenyl)-2-methylacrylate (0.5 g, 2.29 mmol) and diethyltin(IV) dichloride (0.28 g, 1.14 mmol) in dry toluene (100 ml). The reaction mixture was refluxed for 7–8 h, and then allowed to stand overnight. The residual sodium salt was removed by filtration and the solvent was evaporated under reduced pressure leaving a solid residue. Crystals suitable for X-ray analysis were obtained by the recrystallization of the obtained solid residue from a mixture of chloroform/*n*-hexane (4:1) (yield 77%).

### Crystal data

**Table:**

[Sn(C_2_H_5_)_2_(C_10_H_8_ClO_2_)_2_]	*Z* = 2
*M**_r_* = 568.04	*F*_000_ = 572
Triclinic, *P*1	*D*_x_ = 1.564 Mg m^−^^3^
Hall symbol: -P 1	Mo *K*α radiation λ = 0.71073 Å
*a* = 7.5171 (3) Å	Cell parameters from 2981 reflections
*b* = 12.8388 (5) Å	θ = 1.6–26.0º
*c* = 12.8712 (5) Å	µ = 1.31 mm^−^^1^
α = 98.724 (2)º	*T* = 296 (2) K
β = 92.250 (2)º	Prism, colourless
γ = 100.148 (2)º	0.25 × 0.18 × 0.15 mm
*V* = 1205.84 (8) Å^3^	

### Data collection

**Table:**

Bruker KappaAPEXII CCD diffractometer	4718 independent reflections
Radiation source: fine-focus sealed tube	4364 reflections with *I* > 2σ(*I*)
Monochromator: graphite	*R*_int_ = 0.030
Detector resolution: 7.6 pixels mm^-1^	θ_max_ = 26.0º
*T* = 296(2) K	θ_min_ = 1.6º
ω scans	*h* = −9→9
Absorption correction: multi-scan(SADABS; Bruker, 2005)	*k* = −15→15
*T*_min_ = 0.756, *T*_max_ = 0.819	*l* = −15→15
20475 measured reflections	

### Refinement

**Table:**

Refinement on *F*^2^	Secondary atom site location: difference Fourier map
Least-squares matrix: full	Hydrogen site location: inferred from neighbouring sites
*R*[*F*^2^ > 2σ(*F*^2^)] = 0.027	H-atom parameters constrained
*wR*(*F*^2^) = 0.090	*w* = 1/[σ^2^(*F*_o_^2^) + (0.0511*P*)^2^ + 0.4634*P*] where *P* = (*F*_o_^2^ + 2*F*_c_^2^)/3
*S* = 1.23	(Δ/σ)_max_ = 0.002
4718 reflections	Δρ_max_ = 1.19 e Å^−^^3^
281 parameters	Δρ_min_ = −0.70 e Å^−^^3^
Primary atom site location: structure-invariant direct methods	Extinction correction: none

### Special details

**Table:**

Geometry. All esds (except the esd in the dihedral angle between two l.s. planes) are estimated using the full covariance matrix. The cell esds are taken into account individually in the estimation of esds in distances, angles and torsion angles; correlations between esds in cell parameters are only used when they are defined by crystal symmetry. An approximate (isotropic) treatment of cell esds is used for estimating esds involving l.s. planes.
Refinement. Refinement of F^2^ against ALL reflections. The weighted R-factor wR and goodness of fit S are based on F^2^, conventional R-factors R are based on F, with F set to zero for negative F^2^. The threshold expression of F^2^ > 2sigma(F^2^) is used only for calculating R-factors(gt) etc. and is not relevant to the choice of reflections for refinement. R-factors based on F^2^ are statistically about twice as large as those based on F, and R- factors based on ALL data will be even larger.

### Fractional atomic coordinates and isotropic or equivalent isotropic displacement parameters (Å^2^)

**Table:**

	*x*	*y*	*z*	*U*_iso_*/*U*_eq_	
Sn	0.50876 (2)	0.902092 (13)	0.344408 (13)	0.03652 (9)	
Cl1	0.66380 (16)	0.66005 (12)	−0.40308 (7)	0.0845 (4)	
Cl2	−0.09702 (18)	0.43808 (10)	0.81556 (10)	0.0902 (4)	
O1	0.5016 (3)	0.79188 (16)	0.20149 (16)	0.0478 (5)	
O2	0.6318 (4)	0.95295 (18)	0.17974 (18)	0.0543 (6)	
O3	0.3643 (3)	0.76594 (16)	0.40138 (16)	0.0457 (5)	
O4	0.4056 (3)	0.90052 (16)	0.53109 (17)	0.0474 (5)	
C1	0.2876 (5)	0.9737 (3)	0.3070 (3)	0.0521 (8)	
H1A	0.2122	0.9774	0.3664	0.063*	
H1B	0.3333	1.0466	0.2964	0.063*	
C2	0.1719 (5)	0.9147 (4)	0.2096 (3)	0.0667 (10)	
H2A	0.0636	0.9440	0.2038	0.100*	
H2B	0.1402	0.8400	0.2150	0.100*	
H2C	0.2385	0.9226	0.1483	0.100*	
C3	0.7761 (5)	0.9039 (3)	0.4028 (3)	0.0589 (9)	
H3A	0.8583	0.9571	0.3733	0.071*	
H3B	0.7850	0.9255	0.4786	0.071*	
C4	0.8352 (7)	0.7972 (4)	0.3775 (5)	0.0894 (15)	
H4A	0.9571	0.8032	0.4061	0.134*	
H4B	0.8297	0.7760	0.3025	0.134*	
H4C	0.7563	0.7443	0.4079	0.134*	
C5	0.5747 (4)	0.8565 (2)	0.1415 (2)	0.0406 (6)	
C6	0.5873 (5)	0.8162 (2)	0.0280 (2)	0.0427 (7)	
C7	0.6717 (7)	0.8984 (3)	−0.0344 (3)	0.0737 (12)	
H7A	0.6825	0.9685	0.0065	0.111*	
H7B	0.5973	0.8939	−0.0979	0.111*	
H7C	0.7898	0.8857	−0.0521	0.111*	
C8	0.5194 (4)	0.7132 (3)	−0.0069 (2)	0.0423 (6)	
H8	0.4679	0.6757	0.0442	0.051*	
C9	0.5114 (4)	0.6485 (3)	−0.1115 (2)	0.0455 (7)	
C10	0.5829 (5)	0.6840 (3)	−0.2001 (2)	0.0511 (8)	
H10	0.6380	0.7553	−0.1970	0.061*	
C11	0.5719 (5)	0.6128 (4)	−0.2934 (3)	0.0601 (10)	
C12	0.4924 (7)	0.5072 (4)	−0.3012 (3)	0.0790 (14)	
H12	0.4873	0.4605	−0.3644	0.095*	
C13	0.4207 (7)	0.4721 (3)	−0.2139 (3)	0.0803 (13)	
H13	0.3665	0.4006	−0.2177	0.096*	
C14	0.4278 (6)	0.5416 (3)	−0.1206 (3)	0.0587 (9)	
H14	0.3758	0.5166	−0.0626	0.070*	
C15	0.3402 (4)	0.8045 (2)	0.4963 (2)	0.0361 (6)	
C16	0.2341 (4)	0.7320 (2)	0.5609 (2)	0.0348 (5)	
C17	0.1529 (5)	0.6225 (2)	0.5045 (3)	0.0540 (8)	
H17A	0.1480	0.6227	0.4299	0.081*	
H17B	0.0326	0.6021	0.5260	0.081*	
H17C	0.2259	0.5722	0.5213	0.081*	
C18	0.2206 (4)	0.7709 (2)	0.6619 (2)	0.0391 (6)	
H18	0.2753	0.8424	0.6818	0.047*	
C19	0.1352 (4)	0.7213 (2)	0.7467 (2)	0.0402 (6)	
C20	0.0638 (4)	0.6128 (3)	0.7401 (2)	0.0470 (7)	
H20	0.0650	0.5662	0.6775	0.056*	
C21	−0.0091 (5)	0.5743 (3)	0.8270 (3)	0.0565 (9)	
C22	−0.0125 (5)	0.6390 (4)	0.9205 (3)	0.0652 (10)	
H22	−0.0614	0.6114	0.9780	0.078*	
C23	0.0583 (5)	0.7464 (4)	0.9278 (3)	0.0671 (10)	
H23	0.0569	0.7920	0.9911	0.081*	
C24	0.1312 (5)	0.7877 (3)	0.8426 (3)	0.0540 (8)	
H24	0.1782	0.8606	0.8492	0.065*	

### Atomic displacement parameters (Å^2^)

**Table:**

	*U*^11^	*U*^22^	*U*^33^	*U*^12^	*U*^13^	*U*^23^
Sn	0.04917 (14)	0.03108 (12)	0.02580 (12)	−0.00113 (8)	0.00705 (8)	0.00225 (8)
Cl1	0.0831 (7)	0.1456 (11)	0.0321 (4)	0.0466 (7)	0.0138 (4)	0.0048 (5)
Cl2	0.1093 (9)	0.0813 (7)	0.0767 (7)	−0.0200 (6)	0.0012 (6)	0.0474 (6)
O1	0.0790 (15)	0.0352 (10)	0.0261 (10)	0.0024 (10)	0.0124 (10)	0.0018 (8)
O2	0.0764 (16)	0.0401 (12)	0.0402 (12)	−0.0021 (10)	0.0138 (11)	−0.0007 (9)
O3	0.0634 (13)	0.0367 (10)	0.0321 (11)	−0.0046 (9)	0.0106 (9)	0.0043 (8)
O4	0.0638 (13)	0.0315 (10)	0.0405 (12)	−0.0086 (9)	0.0062 (10)	0.0047 (9)
C1	0.061 (2)	0.0546 (18)	0.0385 (17)	0.0111 (15)	−0.0012 (14)	0.0024 (14)
C2	0.063 (2)	0.088 (3)	0.045 (2)	0.000 (2)	−0.0004 (17)	0.0129 (19)
C3	0.0510 (19)	0.067 (2)	0.050 (2)	0.0071 (16)	−0.0010 (15)	−0.0108 (17)
C4	0.073 (3)	0.081 (3)	0.122 (4)	0.019 (2)	0.011 (3)	0.036 (3)
C5	0.0529 (17)	0.0384 (15)	0.0306 (14)	0.0095 (12)	0.0083 (12)	0.0036 (12)
C6	0.0586 (18)	0.0459 (16)	0.0265 (14)	0.0158 (13)	0.0098 (12)	0.0062 (12)
C7	0.126 (4)	0.051 (2)	0.046 (2)	0.010 (2)	0.037 (2)	0.0112 (16)
C8	0.0534 (17)	0.0437 (16)	0.0294 (14)	0.0123 (13)	0.0042 (12)	0.0003 (12)
C9	0.0540 (18)	0.0541 (18)	0.0301 (15)	0.0211 (14)	−0.0025 (12)	0.0004 (13)
C10	0.0588 (19)	0.067 (2)	0.0301 (15)	0.0242 (16)	0.0020 (13)	0.0016 (14)
C11	0.063 (2)	0.091 (3)	0.0299 (16)	0.038 (2)	0.0002 (15)	−0.0032 (17)
C12	0.111 (4)	0.083 (3)	0.043 (2)	0.046 (3)	−0.011 (2)	−0.018 (2)
C13	0.125 (4)	0.057 (2)	0.055 (2)	0.026 (2)	−0.015 (2)	−0.0098 (19)
C14	0.084 (3)	0.0500 (19)	0.0409 (18)	0.0174 (17)	−0.0077 (17)	0.0008 (14)
C15	0.0410 (14)	0.0333 (13)	0.0320 (14)	0.0003 (11)	0.0025 (11)	0.0066 (11)
C16	0.0393 (14)	0.0300 (12)	0.0328 (14)	−0.0005 (10)	0.0028 (11)	0.0053 (10)
C17	0.076 (2)	0.0369 (15)	0.0392 (17)	−0.0146 (14)	0.0138 (16)	0.0004 (13)
C18	0.0462 (15)	0.0331 (13)	0.0341 (15)	−0.0012 (11)	0.0006 (12)	0.0037 (11)
C19	0.0386 (14)	0.0491 (16)	0.0308 (14)	0.0019 (12)	0.0010 (11)	0.0073 (12)
C20	0.0544 (18)	0.0515 (17)	0.0332 (15)	−0.0008 (14)	0.0002 (13)	0.0134 (13)
C21	0.0504 (18)	0.072 (2)	0.049 (2)	−0.0015 (16)	−0.0001 (15)	0.0309 (17)
C22	0.056 (2)	0.102 (3)	0.0405 (19)	0.005 (2)	0.0084 (15)	0.031 (2)
C23	0.069 (2)	0.099 (3)	0.0312 (17)	0.012 (2)	0.0082 (16)	0.0060 (18)
C24	0.0563 (19)	0.062 (2)	0.0388 (17)	0.0046 (16)	0.0042 (14)	0.0010 (15)

### Geometric parameters (Å, °)

**Table:**

Sn—C1	2.110 (3)	C8—C9	1.464 (4)
Sn—C3	2.113 (4)	C8—H8	0.9300
Sn—O3	2.1342 (19)	C9—C10	1.385 (5)
Sn—O1	2.137 (2)	C9—C14	1.391 (5)
Sn—O2	2.477 (2)	C10—C11	1.385 (5)
Sn—O4	2.556 (2)	C10—H10	0.9300
Cl1—C11	1.740 (4)	C11—C12	1.368 (7)
Cl2—C21	1.739 (4)	C12—C13	1.368 (7)
O1—C5	1.286 (4)	C12—H12	0.9300
O2—C5	1.253 (4)	C13—C14	1.376 (5)
O3—C15	1.279 (3)	C13—H13	0.9300
O4—C15	1.251 (3)	C14—H14	0.9300
C1—C2	1.516 (5)	C15—C16	1.489 (4)
C1—H1A	0.9700	C16—C18	1.334 (4)
C1—H1B	0.9700	C16—C17	1.494 (4)
C2—H2A	0.9600	C17—H17A	0.9600
C2—H2B	0.9600	C17—H17B	0.9600
C2—H2C	0.9600	C17—H17C	0.9600
C3—C4	1.507 (6)	C18—C19	1.460 (4)
C3—H3A	0.9700	C18—H18	0.9300
C3—H3B	0.9700	C19—C20	1.390 (4)
C4—H4A	0.9600	C19—C24	1.394 (4)
C4—H4B	0.9600	C20—C21	1.384 (4)
C4—H4C	0.9600	C20—H20	0.9300
C5—C6	1.486 (4)	C21—C22	1.358 (6)
C6—C8	1.333 (5)	C22—C23	1.375 (6)
C6—C7	1.491 (5)	C22—H22	0.9300
C7—H7A	0.9600	C23—C24	1.380 (5)
C7—H7B	0.9600	C23—H23	0.9300
C7—H7C	0.9600	C24—H24	0.9300
			
C1—Sn—C3	154.28 (15)	C6—C8—C9	131.5 (3)
C1—Sn—O3	98.88 (12)	C6—C8—H8	114.3
C3—Sn—O3	101.22 (13)	C9—C8—H8	114.3
C1—Sn—O1	98.94 (11)	C10—C9—C14	118.0 (3)
C3—Sn—O1	99.02 (12)	C10—C9—C8	125.7 (3)
O3—Sn—O1	83.85 (8)	C14—C9—C8	116.3 (3)
C1—Sn—O2	86.19 (12)	C11—C10—C9	119.6 (4)
C3—Sn—O2	88.69 (13)	C11—C10—H10	120.2
O3—Sn—O2	139.87 (8)	C9—C10—H10	120.2
O1—Sn—O2	56.10 (7)	C12—C11—C10	122.0 (4)
C1—Sn—O4	89.58 (11)	C12—C11—Cl1	119.6 (3)
C3—Sn—O4	89.11 (12)	C10—C11—Cl1	118.4 (4)
O3—Sn—O4	54.58 (7)	C11—C12—C13	118.5 (4)
O1—Sn—O4	138.42 (7)	C11—C12—H12	120.8
O2—Sn—O4	165.46 (7)	C13—C12—H12	120.8
C5—O1—Sn	99.75 (17)	C12—C13—C14	120.7 (4)
C5—O2—Sn	84.87 (18)	C12—C13—H13	119.7
C15—O3—Sn	102.54 (16)	C14—C13—H13	119.7
C15—O4—Sn	83.54 (17)	C13—C14—C9	121.2 (4)
C2—C1—Sn	113.8 (3)	C13—C14—H14	119.4
C2—C1—H1A	108.8	C9—C14—H14	119.4
Sn—C1—H1A	108.8	O4—C15—O3	119.3 (2)
C2—C1—H1B	108.8	O4—C15—C16	122.7 (3)
Sn—C1—H1B	108.8	O3—C15—C16	118.0 (2)
H1A—C1—H1B	107.7	C18—C16—C15	117.4 (2)
C1—C2—H2A	109.5	C18—C16—C17	126.9 (3)
C1—C2—H2B	109.5	C15—C16—C17	115.7 (2)
H2A—C2—H2B	109.5	C16—C17—H17A	109.5
C1—C2—H2C	109.5	C16—C17—H17B	109.5
H2A—C2—H2C	109.5	H17A—C17—H17B	109.5
H2B—C2—H2C	109.5	C16—C17—H17C	109.5
C4—C3—Sn	113.5 (3)	H17A—C17—H17C	109.5
C4—C3—H3A	108.9	H17B—C17—H17C	109.5
Sn—C3—H3A	108.9	C16—C18—C19	131.5 (3)
C4—C3—H3B	108.9	C16—C18—H18	114.2
Sn—C3—H3B	108.9	C19—C18—H18	114.2
H3A—C3—H3B	107.7	C20—C19—C24	117.8 (3)
C3—C4—H4A	109.5	C20—C19—C18	125.0 (3)
C3—C4—H4B	109.5	C24—C19—C18	117.1 (3)
H4A—C4—H4B	109.5	C21—C20—C19	119.9 (3)
C3—C4—H4C	109.5	C21—C20—H20	120.1
H4A—C4—H4C	109.5	C19—C20—H20	120.1
H4B—C4—H4C	109.5	C22—C21—C20	122.2 (3)
O2—C5—O1	119.2 (3)	C22—C21—Cl2	119.3 (3)
O2—C5—C6	121.2 (3)	C20—C21—Cl2	118.4 (3)
O1—C5—C6	119.6 (3)	C21—C22—C23	118.3 (3)
C8—C6—C5	117.4 (3)	C21—C22—H22	120.9
C8—C6—C7	127.5 (3)	C23—C22—H22	120.9
C5—C6—C7	115.1 (3)	C22—C23—C24	121.0 (4)
C6—C7—H7A	109.5	C22—C23—H23	119.5
C6—C7—H7B	109.5	C24—C23—H23	119.5
H7A—C7—H7B	109.5	C23—C24—C19	120.8 (4)
C6—C7—H7C	109.5	C23—C24—H24	119.6
H7A—C7—H7C	109.5	C19—C24—H24	119.6
H7B—C7—H7C	109.5		
			
C1—Sn—O1—C5	77.4 (2)	O1—C5—C6—C7	179.1 (3)
C3—Sn—O1—C5	−84.1 (2)	C5—C6—C8—C9	−179.2 (3)
O3—Sn—O1—C5	175.5 (2)	C7—C6—C8—C9	2.6 (6)
O2—Sn—O1—C5	−1.84 (18)	C6—C8—C9—C10	2.1 (6)
O4—Sn—O1—C5	176.96 (16)	C6—C8—C9—C14	−179.4 (3)
C1—Sn—O2—C5	−101.6 (2)	C14—C9—C10—C11	−1.0 (5)
C3—Sn—O2—C5	103.7 (2)	C8—C9—C10—C11	177.4 (3)
O3—Sn—O2—C5	−2.3 (3)	C9—C10—C11—C12	−0.2 (5)
O1—Sn—O2—C5	1.86 (18)	C9—C10—C11—Cl1	−179.8 (2)
O4—Sn—O2—C5	−175.0 (3)	C10—C11—C12—C13	0.6 (6)
C1—Sn—O3—C15	−81.7 (2)	Cl1—C11—C12—C13	−179.8 (3)
C3—Sn—O3—C15	82.2 (2)	C11—C12—C13—C14	0.2 (7)
O1—Sn—O3—C15	−179.83 (19)	C12—C13—C14—C9	−1.5 (7)
O2—Sn—O3—C15	−176.35 (16)	C10—C9—C14—C13	1.9 (5)
O4—Sn—O3—C15	1.39 (17)	C8—C9—C14—C13	−176.8 (4)
C1—Sn—O4—C15	99.8 (2)	Sn—O4—C15—O3	2.2 (3)
C3—Sn—O4—C15	−105.9 (2)	Sn—O4—C15—C16	−177.8 (3)
O3—Sn—O4—C15	−1.40 (17)	Sn—O3—C15—O4	−2.7 (3)
O1—Sn—O4—C15	−3.2 (2)	Sn—O3—C15—C16	177.3 (2)
O2—Sn—O4—C15	172.8 (3)	O4—C15—C16—C18	−4.6 (4)
C3—Sn—C1—C2	146.1 (3)	O3—C15—C16—C18	175.4 (3)
O3—Sn—C1—C2	−72.8 (3)	O4—C15—C16—C17	175.1 (3)
O1—Sn—C1—C2	12.3 (3)	O3—C15—C16—C17	−4.8 (4)
O2—Sn—C1—C2	67.1 (3)	C15—C16—C18—C19	−177.1 (3)
O4—Sn—C1—C2	−126.8 (3)	C17—C16—C18—C19	3.2 (6)
C1—Sn—C3—C4	−168.7 (3)	C16—C18—C19—C20	8.0 (5)
O3—Sn—C3—C4	50.5 (3)	C16—C18—C19—C24	−174.4 (3)
O1—Sn—C3—C4	−34.9 (3)	C24—C19—C20—C21	0.4 (5)
O2—Sn—C3—C4	−90.3 (3)	C18—C19—C20—C21	178.0 (3)
O4—Sn—C3—C4	104.1 (3)	C19—C20—C21—C22	−0.6 (5)
Sn—O2—C5—O1	−2.9 (3)	C19—C20—C21—Cl2	180.0 (2)
Sn—O2—C5—C6	176.1 (3)	C20—C21—C22—C23	0.4 (6)
Sn—O1—C5—O2	3.5 (3)	Cl2—C21—C22—C23	179.8 (3)
Sn—O1—C5—C6	−175.6 (2)	C21—C22—C23—C24	−0.1 (6)
O2—C5—C6—C8	−178.4 (3)	C22—C23—C24—C19	−0.1 (6)
O1—C5—C6—C8	0.6 (5)	C20—C19—C24—C23	−0.1 (5)
O2—C5—C6—C7	0.0 (5)	C18—C19—C24—C23	−177.9 (3)

### Hydrogen-bond geometry (Å, °)

**Table:**

*D*—H···*A*	*D*—H	H···*A*	*D*···*A*	*D*—H···*A*
C7—H7A···O2	0.96	2.31	2.780 (5)	109
C8—H8···O1	0.93	2.30	2.736 (3)	108
C17—H17A···O3	0.96	2.31	2.749 (4)	107
C18—H18···O4	0.93	2.37	2.785 (3)	107
